# Supplementary material for: Compliance with smoke-free legislation in public places: An observational study in a northeast city of Bangladesh
Source: PLoS One. 2023 Apr 26;18(4):e0283650. doi: 10.1371/journal.pone.0283650 (PMC10132694; doi:10.1371/journal.pone.0283650)
Supplement: S1 File — (DOCX) [file pone.0283650.s001.docx]

**S1 Materials (Data Collection Form)**

| **ID** |  |
| --- | --- |

| **PART A. LOCATION INFORMATION** | | | | | | | |
| --- | --- | --- | --- | --- | --- | --- | --- |
| 1. Name of location / facility | | | | |  | | |
| 1. Address |  | | | | | | |
| 1. Total number of buildings at location | | | | | | |  |
| 1. **Type of location (choose only one)** | | | | | | | |
| 1. Educational institution (primary & secondary) | | | | | | | 15. Industry/Factory/Indoor workplace |
| 1. Educational institution (college & university) | | | | | | | 16. Residential Hotel |
| 1. Library | | | | | | | 17. Court building |
| 1. Hospital and clinic building | | | | | | | 18. Airport building |
| 1. Cinema hall, exhibition center, theatre hall | | | | | | | 19. Sea port building |
| 1. Restaurants surrounded by wall in all sides (single room) | | | | | | | 20. River-port building |
| 1. Children park | | | | | | | 21. Railway station building |
| 1. Fitness center/Sports facility (covered places) | | | | | | | 22. Bus terminal building |
| 1. Restaurants surrounded by wall in all sides (more than one room) | | | | | | | 23. Shopping center |
| 1. Fitness center/Sports facility (uncovered places) | | | | | | | 24. Public toilet |
| 1. Government office | | | | | | | 25. Fairs |
| 1. Semi-government office | | | | | | | 26. Designated queues or places for passengers waiting to ride on public transports (পাবলিক পরিবহণে আরোহণের জন্য অপেক্ষায় থাকা যাত্রীদের মনোনীত স্থান বা নির্দিষ্ট সারি) |
| 1. Autonomous office | | | | | | | 27. Any other public area to be combinedly used by the general people or, any or all places declared time to time by the government or local government organization by a general or special order (জনসাধারণ কর্তৃক সম্মিলিতভাবে ব্যবহার্য্য অন্য কোন স্থান অথবা সরকার বা স্থানীয় সরকার প্রতিষ্ঠান কর্তৃক, সাধারণ বা বিশেষ আদেশ দ্বারা সময় সময় ঘোষিত অন্য যে কোন বা সকল স্থান) |
| 1. Private office | | | | | | |  |
| 1. Date of visit | | |  | | | | |
| 1. Data collector code | | | | | |  | |
| 1. Time of entry to location _______________am / pm | | | | | | | |
| 1. Time of departure _______________am / pm | | | | | | | |
| 1. Photo taken? | | | |  1= YES  2= NO | | | |
| 1. Result of observation | | | | | |  1= Finished  2= Not Finished **(Go to Q11)** | |
| 1. If observation not finished, reason why: | |  1= Data collector not allowed to enter building/location   2= Building/location out of business   3= Other: __________________ | | | | | |
| **Note: Write here, if any abnormalities are found regarding the observation of any question.** | | | | | | | |

| **PART B. OBSERVATION INFORMATION** | | | |
| --- | --- | --- | --- |
| **Observation Indoors (where indoor designated smoking areas are not permitted – যেখানে ধূমপানের জন্য নির্ধারিত স্থান রাখার অনুমতি নেই)** | | | |
| 1. Name or number of building |  | | |
| 1. Is anyone smoking tobacco products indoors?   If yes, number of persons smoking __________ | |  1= YES |  2= NO |
| If anyone smoking e-cigarette, number of persons: _________ | | | |
| 1. Do you see a designated smoking area indoors? (Choose “YES” if you see a designated area, even if no one is smoking in it.) | |  1= YES |  2= NO |
| 1. Is there signage stating that smoking is not permitted?   **If no, skip to Q7 and if yes, continue with Q5** | |  1= YES |  2= NO |
| 1. Whether signages are displayed at | |  1= Main entrance |  2= Other conspicuous places |
|  |  |  3= None of these | |
| 1. Does signage comply with the law? | |  1= In contents |  2= In design |
|  |  | 3=In language (μ1=Bangla 2=English) | |
|  |  |  4= In size |  5= None of these |
| 1. Whether some cigarettes buts, bidi ends or ashes are found in this public place? | |  1= YES |  2= NO |
| 1. Whether any of the following smoking aids are in place at the indoor of public place under study? | |  1= Ashtrays |  2= Ashbins |
|  |  |  3= Matchboxes |  4= Lighters |
|  |  |  5=Others ______ |  6= None of these |
| **Note: Write here, if any abnormalities are found regarding the observation of any question.** | | | |

| **PART C. OBSERVATION INFORMATION** | | | |
| --- | --- | --- | --- |
| **Observation Indoors (where indoor designated smoking areas are permitted – যেখানে ধূমপানের জন্য নির্ধারিত স্থান রাখার অনুমতি আছে)** | | | |
| 1. Name or number of building |  | | |
| 1. Do you see a designated smoking area indoors? (Choose “YES” if you see a designated area, even if no one is smoking in it.) | |  1= YES |  2= NO |
| 1. Is anyone smoking tobacco products indoors, other than in a designated smoking area?   If yes, number of persons smoking: __________ | |  1= YES |  2= NO |
| If anyone smoking e-cigarette, number of persons: _________ | | | |
| 1. Is there signage stating that smoking is not permitted?   **If no, skip to Q7 and if yes, continue with Q5** | |  1= YES |  2= NO |
| 1. Whether signages are displayed at | |  1= Main entrance |  2= Other conspicuous places |
|  |  |  3= None of these | |
| 1. Does signage comply with the law? | |  1= In contents |  2= In design |
|  |  | 3=In language (μ1=Bangla 2=English) | |
|  |  |  4= In size |  5= None of these |
| 1. Whether some cigarettes buts, bidi ends or ashes are found in this public place? | |  1= YES |  2= NO |
| 1. Whether any of the following smoking aids are in place at the indoor of public place under study? | |  1= Ashtrays |  2= Ashbins |
|  |  |  3= Matchboxes |  4= Lighters |
|  |  |  5=Others ______ |  6= None of these |
| **Note: Write here, if any abnormalities are found regarding the observation of any question.** | | | |

| **PART D. OBSERVATION INFORMATION** | | | | |
| --- | --- | --- | --- | --- |
| **Observation Outdoors (inside location boundary)** | | | | |
| 1. Is anyone smoking tobacco products anywhere outdoors at the location? (If there are no outdoor grounds at this location, choose “not applicable”)   If yes, number of persons smoking: __________ |  1= YES | |  2= NO   3= Not Applicable | |
| If anyone smoking e-cigarette, number of persons: _________ | | | | |
| 1. Is there signage stating that smoking is not permitted in the grounds of the venue/location?   **If no, skip to Q5 and if yes, continue with Q3** |  1= YES | |  2= NO | |
| 1. Whether signages are displayed at the outdoor of this public place at |  1= Main entrance | |  2= Other conspicuous places | |
|  |  3= None of these | | | |
| 1. Does signage comply with the law? |  1= In contents | |  2= In design | |
|  | 3=In language (μ1=Bangla 2=English) | | | |
|  |  4= In size | |  5= None of these | |
| 1. Whether some cigarettes buts, bidi ends or ashes are found on the outdoor of this public place? |  1= YES | |  2= NO | |
| 1. Whether the following smoking aids are in place at the outdoor of public place under study? |  1= Ashtrays | |  2= Ashbins | |
|  |  3= Matchboxes | |  4= Lighters | |
|  |  5=Others ______ | |  6= None of these | |
| 1. Availability of tobacco (tobacco vendors/shops) within the venue/location (inside boundary)   If yes, type of vendors/shops:   1= Permanent  2= Temporary/mobile |  1= YES | |  2= NO | |
| **Observation Outdoors (outside location boundary)** | | | | |
| 1. Is anyone smoking within 100 meters of the venue/location?   If yes, number of persons: ________ | |  1= YES | |  2= NO |
| If anyone smoking e-cigarette, number of persons: _________ | | | | |
| 1. Availability of tobacco (tobacco vendors/shops) within 100 meters from the venue/location:   If yes, type of vendors/shops:   1= Permanent  2= Temporary/mobile | |  1= YES | |  2= NO |
| **Note: Write here, if any abnormalities are found regarding the observation of any question.** | | | | |
