# Supplementary material for: Compliance with smoke-free legislation in public places: An observational study in a northeast city of Bangladesh
Source: PLoS One. 2023 Apr 26;18(4):e0283650. doi: 10.1371/journal.pone.0283650 (PMC10132694; doi:10.1371/journal.pone.0283650)
Supplement: S2 File — (DOCX) [file pone.0283650.s002.docx]

**S2 Materials**

Table of Contents

[Supplementary Tables 2](#_Toc90930270)

[S1 Table: Distribution of all types of public places in Sylhet City included in the study 2](#_Toc90930271)

[S2 Table: Compliance with specific indicators of smoke-free legislation in different public places in Sylhet City (observation indoors) 3](#_Toc90930272)

[S3 Table: Compliance with specific indicators of smoke-free legislation in different public places in Sylhet City (observation outdoors) 4](#_Toc90930273)

[S4 Table: Compliance of “no smoking signages” with the “Smoking and Tobacco Products Usage (Control) (Amendment) Act” (indoor) 5](#_Toc90930274)

[S5 Table: Compliance of “no smoking signages” with the “Smoking and Tobacco Products Usage (Control) Act” (outdoor) 6](#_Toc90930275)

[S6 Table: Smoking aids were found at the public places under study (indoor) 7](#_Toc90930276)

[S7 Table: Smoking aids were found at the public places under study (outdoor) 7](#_Toc90930277)

# Supplementary Tables

## S1 Table: Distribution of all types of public places in Sylhet City included in the study

| **Name of locations** | **n (%)** |
| --- | --- |
| Library | 3 (0.4) |
| Hospital and clinic building | 90 (13.4) |
| Children park | 2 (0.3) |
| Fitness center/Sports facility (covered places) | 7 (1.0) |
| Restaurants surrounded by wall in all sides (single room) | 125 (18.6) |
| Restaurants surrounded by wall in all sides (more than one room) | 52 (7.7) |
| Fitness center/Sports facility (uncovered places) | 1 (0.1) |
| Government office | 79 (11.7) |
| Private office | 53 (9.7) |
| Industry/Factory/Indoor workplace | 12 (1.8) |
| Residential Hotel | 58 (8.6) |
| Court building | 1 (0.1) |
| Railway station building | 2 (0.3) |
| Bus terminal building | 12 (1.8) |
| Shopping center | 128 (19.0) |
| Public toilet | 5 (0.7) |
| Designated queues or places for passengers waiting to ride on public transports | 20 (3.0) |
| Any other public area to be combinedly used by the general people or, any or all places declared time to time by the government or local government organization by a general or special order | 23 (3.4) |
| **Total** | **673 (100.0)** |

## S2 Table: Compliance with specific indicators of smoke-free legislation in different public places in Sylhet City (observation indoors)

| **Compliance indicators** | **Type of public places** | | | | | | |
| --- | --- | --- | --- | --- | --- | --- | --- |
|  | **Accommodation facilities (n=58)** | **Eateries (n=176) ^a^** | **Offices and workplaces (n=147) ^b^** | **Healthcare facilities (n=90)** | **Transit points (n=14)** | **Most frequently visited other public places (n=150) ^c^** | **All public places (n=635) ^d^** |
| Absence of active smoking, n (%) | 55 (94.8) | 165 (93.7) | 146 (99.3) | 88 (97.7) | 11 (78.5) | 93 (62.0) | 558 (87.9) |
| Absence of a designated smoking area indoors (where not permitted), n (%) | NA | 124 (99.2) | 3 (100.0) | 90 (100.0) | NA | 7 (100.0) | 224 (99.6) |
| Presence of ‘no smoking’ signage, n (%) | 14 (24.1) | 51 (29.0) | 31 (21.1) | 23 (25.6) | 4 (28.6) | 18 (12.0) | 141 (22.2) |
| Display of ‘no smoking’ signage at the main entrance and other conspicuous places, n (%) | 2 (3.4) | 6 (3.4) | 13 (8.8) | 10 (11.1) | 0 (0.0) | 3 (2.0) | 34 (5.4) |
| ‘No smoking’ signage complies with the law, n (%) | 0 (0.0) | 4 (2.3) | 2 (1.4) | 3 (3.3) | 0 (0.0) | 0 (0.0) | 9 (1.4) |
| Absence of cigarettes buts, bidi ends or ashes, n (%) | 40 (69.0) | 144 (81.8) | 108 (73.5) | 67 (74.4) | 6 (42.9) | 43 (28.7) | 408 (64.3) |
| Absence of smoking aids such as ashtrays, ashbins, matchboxes, lighters, n (%) | 48 (82.8) | 171 (97.2) | 140 (95.2) | 88 (97.8) | 9 (64.3) | 105 (70.0) | 561 (88.3) |
| **Good compliance, n (%)** | 2 (3.4) | 38 (21.6) | 9 (6.1) | 20 (22.2) | 0 (0.0) | 1 (0.7) | 70 (11.0) |
| **Moderate compliance, n (%)** | 39 (67.2) | 122 (69.3) | 107 (72.8) | 68 (75.6) | 5 (35.7) | 47 (31.3) | 388 (61.1) |
| **Poor compliance, n (%)** | 17 (29.3) | 16 (9.1) | 31 (21.1) | 2 (2.2) | 9 (64.3) | 102 (68.0) | 177 (27.9) |
| **Total compliance* (%)** | 45.7 | 58.1 | 57.0 | 58.6 | 35.7 | 39.2 | 52.7 |

*a: DSA permit, n=51, DSA not permit, n=125*

*b: DSA permit, n=144, DSA not permit, n=3*

*c: DSA permit, n=143, DSA not permit, n=7*

*d: DSA permit, n=338, DSA not permit, n=135*

*Good compliance: Compliance with 5-7 indicators; Moderate compliance: Compliance with 3-4 indicators; Poor compliance: Compliance with 0-2 indicators*

** Total compliance for each category of public places was calculated by averaging the percentages of various compliance indicators.*

## S3 Table: Compliance with specific indicators of smoke-free legislation in different public places in Sylhet City (observation outdoors)

| **Compliance indicators** | **Type of public places** | | | | | | |
| --- | --- | --- | --- | --- | --- | --- | --- |
|  | **Accommodation facilities, (n= 8)** | **Eateries (n= 53)** | **Offices and workplaces (n= 110)** | **Healthcare facilities (n=47)** | **Transit points (n=31)** | **Most frequently visited other places (n=64)** | **All public places (n=313)** |
| Absence of active smoking, n (%) | 7 (87.5) | 34 (64.2) | 95 (86.4) | 37 (78.7) | 2 (6.5) | 20 (31.3) | 195 (62.3) |
| Presence of ‘no smoking’ signage, n (%) | 0 (0.0) | 4 (7.5) | 20 (18.2) | 6 (12.8) | 0 (0.0) | 3 (4.7) | 33 (10.5) |
| Display of ‘no smoking’ signage at the main entrance and other conspicuous places, n (%) | 0 (0.0) | 1 (1.8) | 10 (9) | 0 (0.0) | 0 (0.0) | 1 (1.5) | 12 (3.8) |
| ‘No smoking’ signage complies with the law, n (%) | 0 (0.0) | 4 (2.3) | 2 (1.8) | 1 (2.1) | 0 (0.0) | 0 (0.0) | 7 (2.2) |
| Absence of cigarettes buts, bidi ends or ashes, n (%) | 0 (0.0) | 15 (28.3) | 29 (26.4) | 9 (19.1) | 0 (0.0) | 4 (6.3) | 57 (18.2) |
| Absence of smoking aids such as ashtrays, ashbins, matchboxes, lighters, n (%) | 3 (37.5) | 32 (60.4) | 89 (80.9) | 39 (83.0) | 2 (6.5) | 28 (43.7) | 193 (61.7) |
| **Good compliance, n (%)** | 0 (0.0) | 0 (0.0) | 4 (3.6) | 0 (0.0) | 0 (0.0) | 1 (1.6) | 5 (1.6) |
| **Moderate compliance, n (%)** | 0 (0.0) | 17 (32.1) | 33 (30.0) | 9 (19.1) | 0 (0.0) | 4 (6.3) | 63 (20.1) |
| **Poor compliance, n (%)** | 8 (100) | 36 (67.9) | 73 (66.4) | 38 (80.9) | 31 (100) | 59 (92.2) | 245 (78.3) |
| **Total compliance* (%)** | 20.8 | 27.4 | 37.1 | 32.6 | 2.2 | 14.6 | 26.5 |

*Good compliance: Compliance with 5-6 indicators; Moderate compliance: Compliance with 3-4 indicators; Poor compliance: Compliance with 0-2 indicators*

** Total compliance for each category of public places was calculated by averaging the percentages of various compliance indicators.*

## S4 Table: Compliance of “no smoking signages” with the “Smoking and Tobacco Products Usage (Control) (Amendment) Act” (indoor)

| **Variables** | **All public places, n=141** | **Accommodation facilities, n=14** | **Eateries, n=51** | **Offices and workplaces, n=31** | **Healthcare facilities, n=23** | **Most frequently visited places, n=18** | **Transit points, n=4** |
| --- | --- | --- | --- | --- | --- | --- | --- |
| **Signage displayed at*, n (%)** | | | | | | | |
| Both main entrance and other conspicuous places | 34 (24.1) | 2 (14.3) | 6 (11.8) | 13 (41.9) | 10 (43.5) | 3 (16.7) | 0 (0.0) |
| Main entrance | 75 (53.2) | 9 (64.3) | 20 (39.2) | 23 (74.2) | 15 (65.2) | 6 (33.3) | 2 (50.0) |
| Other conspicuous places | 95 (67.4) | 7 (50.0) | 35 (68.6) | 21 (67.7) | 17 (73.9) | 13 (72.2) | 2 (50.0) |
| **Signage complies with the law*, n (%)** | | | | | | | |
| In contents | 29 (20.6) | 5 (35.7) | 12 (23.5) | 3 (9.7) | 9 (39.1) | 0 (0.0) | 0 (0.0) |
| In design | 60 (42.6) | 7 (50.0) | 25 (49.0) | 10 (32.3) | 13 (56.5) | 2 (11.1) | 3 (75.0) |
| In language (Bangla) | 100 (70.9) | 8 (57.1) | 38 (74.5) | 24 (77.4) | 15 (65.2) | 11 (61.1) | 4 (100.0) |
| In language (English) | 78 (55.3) | 13 (92.9) | 28 (54.9) | 13 (41.9) | 15 (65.2) | 8 (44.4) | 1 (25.0) |
| In language (Both Bangla and English) | 42 (29.8) | 7 (50.0) | 17 (33.3) | 6 (19.4) | 9 (39.1) | 2 (11.1) | 1 (25.0) |
| In size | 25 (17.7) | 3 (21.4) | 10 (19.6) | 4 (12.9) | 6 (26.1) | 2 (11.1) | 0 (0.0) |
| All of the above | 9 (6.4) | 0 (0.0) | 4 (7.8) | 2 (6.5) | 3 (13.0) | 0 (0.0) | 0 (0.0) |

** Note: Multiple responses.*

## S5 Table: Compliance of “no smoking signages” with the “Smoking and Tobacco Products Usage (Control) (Amendment) Act” (outdoor)

| **Variables** | **All public places, n=33** | **Accommodation facilities, n=0** | **Eateries, n=4** | **Offices and workplaces, n=20** | **Healthcare facilities, n=6** | **Most frequently visited places, n=3** | **Transit points, n=0** |
| --- | --- | --- | --- | --- | --- | --- | --- |
| **Signage displayed at*, n (%)** | | | | | | | |
| Both main entrance and other conspicuous places | 12 (36.4) | 0 (0.0) | 1 (25.0) | 10 (50.0) | 0 (0.0) | 1 (33.3) | 0 (0.0) |
| Main entrance | 27 (81.8) | 0 (0.0) | 3 (75.0) | 19 (95.0) | 2 (33.3) | 3 (100.0) | 0 (0.0) |
| Other conspicuous places | 18 (54.5) | 0 (0.0) | 2 (50.0) | 11 (55.0) | 4 (66.7) | 1 (33.3) | 0 (0.0) |
| **Signage complies with the law*, n (%)** | | | | | | | |
| In contents | 8 (24.2) | 0 (0.0) | 1 (25.0) | 3 (15.0) | 4 (66.7) | 0 (0.0) | 0 (0.0) |
| In design | 12 (36.4) | 0 (0.0) | 2 (50.0) | 6 (30.0) | 3 (50.0) | 1 (33.3) | 0 (0.0) |
| In language (Bangla) | 27 (81.8) | 0 (0.0) | 4 (100.0) | 17 (85.0) | 5 (83.3) | 1 (33.3) | 0 (0.0) |
| In language (English) | 14 (42.4) | 0 (0.0) | 0 (0.0) | 7 (35.0) | 5 (83.3) | 2 (66.7) | 0 (0.0) |
| In language (Both Bangla and English) | 8 (24.2) | 0 (0.0) | 0 (0.0) | 4 (20.0) | 4 (66.7) | 0 (0.0) | 0 (0.0) |
| In size | 6 (18.2) | 0 (0.0) | 3 (75.0) | 2 (10.0) | 1 (16.7) | 0 (0.0) | 0 (0.0) |
| All of the above | 3 (9.1) | 0 (0.0) | 0 (0.0) | 2 (10.0) | 1 (16.7) | 0 (0.0) | 0 (0.0) |

** Note: Multiple responses.*

## S6 Table: Smoking aids were found at the public places under study (indoor)

| **Variables** | **All public places, n=74** | **Accommodation facilities, n=10** | **Eateries, n=5** | **Offices and workplaces, n=7** | **Healthcare facilities, n=2** | **Most frequently visited places, n=45** | **Transit points, n=5** |
| --- | --- | --- | --- | --- | --- | --- | --- |
| **Name of smoking aids*, n (%)** | | | | | | | |
| Ashtrays | 7 (9.5) | 3 (30.0) | 0 (0.0) | 4 (57.1) | 0 (0.0) | 0 (0.0) | 0 (0.0) |
| Ashbins | 10 (13.5) | 3 (30.0) | 0 (0.0) | 0 (0.0) | 0 (0.0) | 7 (15.6) | 0 (0.0) |
| Matchboxes | 57 (77.0) | 5 (50.0) | 4 (80.0) | 4 (57.1) | 2 (100) | 38 (84.4) | 4 (80.0) |
| Lighters | 52 (70.3) | 4 (40.0) | 3 (60.0) | 1 (14.3) | 2 (100) | 37 (82.2) | 5 (100.0) |
| Others | 1 (1.4) | 0 (0.0) | 0 (0.0) | 0 (0.0) | 0 (0.0) | 1 (2.2) | 0 (0.0) |

** Note: Multiple responses.*

## S7 Table: Smoking aids were found at the public places under study (outdoor)

| **Variables** | **All public places, n=120** | **Accommodation facilities, n=5** | **Eateries, n=21** | **Offices and workplaces, n=21** | **Healthcare facilities, n=8** | **Most frequently visited places, n=36** | **Transit points, n=29** |
| --- | --- | --- | --- | --- | --- | --- | --- |
| **Name of smoking aids*, n (%)** | | | | | | | |
| Ashtrays | 2 (1.7) | 0 (0.0) | 0 (0.0) | 2 (9.5) | 0 (0.0) | 0 (0.0) | 0 (0.0) |
| Ashbins | 4 (3.3) | 2 (40.0) | 0 (0.0) | 1 (4.8) | 0 (0.0) | 1 (2.8) | 0 (0.0) |
| Matchboxes | 111 (92.5) | 3 (60.0) | 20 (11.3) | 15 (71.4) | 8 (100.0) | 36 (100.0) | 29 (100.0) |
| Lighters | 97 (80.8) | 3 (60.0) | 18 (10.2) | 9 (42.9) | 6 (75.0) | 32 (88.9) | 29 (100.0) |

** Note: Multiple responses.*
