# Supplementary material for: Compliance with smoke-free legislation in public places: An observational study in a northeast city of Bangladesh
Source: PLoS One. 2023 Apr 26;18(4):e0283650. doi: 10.1371/journal.pone.0283650 (PMC10132694; doi:10.1371/journal.pone.0283650)
Supplement: Attachment — Submitted filename: Response to Reviewer.docx [file pone.0283650.s004.docx]

**Response to Reviewer**

**Authors’ Responses to Reviewer 1:**

We are giving thanks the reviewer for reviewing this manuscript. The raised points have certainly improved the quality of the manuscript. We have addressed all the comments and made the required changes in our manuscript.

**Review Comment #1:** The study is conducted with lot of efforts and is being presented effectively. The need of study is also justified. Data are collected with lot of care and is presented well. There is one objection on analysis which to me seems of a great concern. The average percentages concept used in the analysis, cannot be considered correct. Few suggestions for overall percentages

1. Summing up all compliance options with OR condition (for instance if for one case {absent of active smoking =1, presence of no smoking signage = 0, Display of no signage at entrance = 0, no smoking signage is in compliance with law = 1, Absence of cigarretes buts, bidi ends or ashes = 1, absence of smoking aids = 0} for overall compliance it will count "1" for that cases) The overall percentage will be calculated through that column of overall. this scenario will work if every option is given equal weight age.

2. If every option has different weightage then maximum of the weightage will be the final option.

3. A criterion can be made (e.g. if 4 of the 7 compliances are answered "yes" and 3 are answered "no", overall it will go to "yes" IF overall percentages are changed by using one of these methods then the manuscript is acceptable in my point of view. If some justification for using average percentages is provided by the author and is acceptable by the editorial board or by some other expert in the field that will be appreciable.

**Reply from the authors:**

Thanks for raising this concern. However, this average percentage concept has been used in several previous research published in high-impact journals, including a recent publication. We have mentioned in the “Statistical Analysis” section of our manuscript that we have used this approach as it has been used in previous research. The references are the followings:

Goel S, Sharma D, Gupta R, Mahajan V. Compliance with smoke-free legislation and smoking behaviour: Observational field study from Punjab, India. Tobacco Control. 2018;27: 407–413. doi:10.1136/tobaccocontrol-2016-053559

Goel S, Ravindra K, Singh RJ, Sharma D. Effective smoke-free policies in achieving a high level of compliance with smoke-free law: Experiences from a district of North India. Tobacco Control. 2014;23: 291–294. doi:10.1136/tobaccocontrol-2012-050673

Basnet LB, Budhathoki SS, Adhikari B, Thapa J, Neupane B, Moses T, et al. Compliance with the smoke-free public places legislation in Nepal: A cross-sectional study from Biratnagar Metropolitan City. PLoS ONE. 2022;17. doi:10.1371/JOURNAL.PONE.0264895

However, we assume that the suggestions of the reviewer are also worthy and it will improve the quality of the reporting of the manuscript. So, based on the reviewer’s suggestions and our consensus, we have also rated the compliance in the following ways:

For indoors, a location was given a rating of ‘good compliance’, ‘moderate compliance’ or ‘poor compliance" depending on the number of indicators it met: 5-7, 3-4, or 0-2 for good compliance, moderate compliance, or poor compliance, respectively. For outdoors, a location was leveled as ‘good compliance’, ‘moderate compliance', or ‘poor compliance’ if the place complied with 5-6 indicators, 3-4 indicators, or 0-2 indicators, respectively.

The interesting fact is that percentage of average/overall compliance, and moderate compliance (using the new approach) are almost identical in most cases.

**Authors’ Responses to Reviewer 2:**

We are giving thanks the reviewer for reviewing this manuscript. The raised points have certainly improved the quality of the manuscript. We have addressed all the comments and made the required changes in our manuscript.

**Review Comment #1:**  I think the paper addresses an interesting and important research question in a country like Bangladesh with a high current smoking prevalence. However, the English in the present manuscript is not of publication quality for Plos One journal and requires improvement. I recommend the manuscript be copy edited by a native English speaker.

**Reply from the authors:**

Thanks for raising this concern. This time, our manuscript has been copy-edited by a native English speaker.

**Review Comment #2:**

**Abstract**
• Please indicate the study period in the abstract. Please mention the place of data collection in the objectives part.

**Reply from the authors:**

We have addressed accordingly.

• The technique of the data collection should be mentioned in the method section of the abstract. What factors were considered during the observation, (e.g. evidence of smoking (observed smoking, cigarette butt litter, and display of ashtrays, the presence of designated smoking areas/rooms, presence of points of sale or etc. ) and what was the outcome of the study?

**Reply from the authors:**

We have addressed accordingly.

• The results section should be limited to the most important numbers. There is no need to bring all detailed results such as % of compliance in each observed section.

**Reply from the authors:**

We have addressed accordingly.

**Review Comment #3:**

**Introduction**
In the introduction part there is huge information on the burden of smoking worldwide. However, there is lacking on evidence on the smoking prevalence and burden of the tobacco use in Bangladesh.

**Reply from the authors:**

We have addressed accordingly, and added the followings:

There is a relatively high prevalence of exposure to SHS in Bangladesh [5]. According to a nationwide survey conducted in Bangladesh, the prevalence of SHS was 43% [9]. The Global Burden of Disease Study estimates that in 2019, tobacco was responsible for around 157,862 fatalities in Bangladesh, which is approximately 19% of all deaths [10]. According to World Health Organization (WHO), each year in Bangladesh, 1.2 million people suffer from diseases that are associated with tobacco use [11]. A study conducted in Bangladesh revealed that in 2018, 61,000 children were suffering from diseases due to exposure to SHS [12]. A ten-year prospective study that included twenty thousand adult participants found that smoking was responsible for 25% of deaths among males and 7.6% of deaths among females [13]. This is clear evidence that Bangladesh has a huge number of deaths and illnesses related to tobacco use, necessitating national attention to this massive issue.

**Methods**

**Review Comment #4:**

Please indicate the reason for the selection of this city to conduct the study.

**Reply from the authors:**

We have addressed accordingly, and added the followings:

There are three major cities in Bangladesh. These are Dhaka, Chattogram, and Sylhet cities. Among these, Sylhet city has been conveniently selected for this study because of its status as a significant metropolitan city in that country.

**Review Comment #5:**

The tool provided by the John Hopkins university is almost a comprehensive tool. Why was it not used alone and researcher decided to modify it through literature review? Which changes have been made to the original instrument? Please describe the changes made in the study tool.

**Reply from the authors:**

We have addressed accordingly, and added the followings:

We made some adjustments to the existing compliance study tool. For example, the indicators "display of 'no smoking' signages at the main entrance and other conspicuous places and ‘no smoking’ signage complies with the law" was taken because these are part of the Smoking and Tobacco Products Usage (Control) (Amendment) Act, 2013, Bangladesh. Additionally, the indicator "absence of cigarettes buts, bidi ends or ashes" was considered based on similar studies as evidence of recent smoking.

**Review Comment #6:**

I have some concerns about the operational definitions too. I found them more conceptual rather than operational. Please bring them in a way that reader realize how you defined variables such as active smoking (for example, active smoking in a public place: Active smoking in a public place was marked as present if anyone was seen smoking during the researcher’s visit at the public place being observed for the study) or other variables such as smoking aids, cigarette/bidi stubs/butts, cigarette smell and etc. This part should be rewritten according to the variables included in the tool.

**Reply from the authors:**

Thanks for your suggestions. We have rewritten this part, and now it stands as follows:

**Operational definitions**

**Compliance:** It is the degree to which the Smoking and Tobacco Products Usage (Control) (Amendment) Act, 2013, Bangladesh, is being obeyed.

**Smoke-free legislation:** The law that prohibits smoking in a specified area, i.e., the Smoking and Tobacco Products Usage (Control) (Amendment) Act, 2013, Bangladesh.

**Public Places:** All the places those are defined as public places in the Smoking and Tobacco Products Usage (Control) (Amendment) Act, 2013, Bangladesh.

**Smoking:** Inhaling or exhaling the smoke of tobacco and also includes keeping or controlling any flamed tobacco products.

**Active smoking:** Active smoking in a public place was marked as present if anyone was seen smoking during the researcher’s visit at the public place being observed for the study.

**Designated smoking area (DSA):** The section of a public place that has been set apart and designated as a smoking area by the owner, caretaker, manager, or other individual in charge of the public place in which smoking is permitted. DSA was marked as present if it was noticed during the researcher’s visit at the public place where it is not permitted to be placed.

**‘No smoking’ signage:** According to the Smoking and Tobacco Products Usage (Control) (Amendment) Act, 2013, every public place shall arrange to display a caution notice **"Refrain from Smoking, It is a** **Punishable Offence"** in Bengali and in English language at the entrance and in one or more places inside a public place. The size of a caution notice board in a public place shall be at least 40 centimeters × 20 centimeters. Every public place shall arrange to display the caution notice in red letters against a white background or in white letters against a red background with a no-smoking sign.

**Cigarette buts, bidi ends, or ashes:** Cigarette buts, bidi ends, or ashes were marked as present as evidence of previous smoking in a particular public place if any of these was noticed during the researcher’s visit at the public place being observed for the study.

**Smoking aids:** According to the Smoking and Tobacco Products Usage (Control) (Amendment) Act, 2013, no smoking aids such as ashtrays, ashbins, matchboxes, lighters can be kept in the smoke-free area.

**Discussion**

**Review Comment #7:**

Please compare you results with other developing countries or countries close by like Pakistan, India or Nepal.

**Reply from the authors:**

Thanks for your suggestions. We compared our results with a bunch of articles from India. This time, we have compared our findings with a few articles from Pakistan, Nepal, and India also. We added the following references, and changes can be found in the track changes in the manuscript:

Basnet LB, Budhathoki SS, Adhikari B, Thapa J, Neupane B, Moses T, et al. Compliance with the smoke-free public places legislation in Nepal: A cross-sectional study from Biratnagar Metropolitan City. PLoS ONE. 2022;17. doi:10.1371/JOURNAL.PONE.0264895

Ahsan H, Hoe C, Aslam F, Wright K, Cohen J, Kennedy R. Compliance with smoke-free policies at indoor and outdoor public places: an observational study in Pakistan. Eastern Mediterranean health journal. 2022;28: 50–57. doi:10.26719/EMHJ.22.005

Banandur P, Kumar M, Gopalakrishna G. Awareness and compliance to anti-smoking law in South Bengaluru, India. Tobacco Prevention & Cessation. 2017;3. doi:10.18332/tpc/76549

**Review Comment #8:**

It is better to mention the name of the country instead of the name of the authors for similar studies that actually mentioned in the discussion part.

**Reply from the authors:**

We have addressed accordingly.

**Review Comment #9:**

I suggest that at the end of the discussion part the authors elaborate potential solutions and policy options to address low compliance rate. What is your suggestion about the Venue managers? Are they responsible for ensuring that all public places that they operate are smoke free? What can be done to increase population awareness regarding the harm of smoking (social media, tobacco campaign,...)?

**Reply from the authors:**

Thanks for your suggestions. We have addressed accordingly, and added the followings:

When viewed from the perspective of a developing nation such as Bangladesh, the overall barriers to the successful implementation of tobacco control policies could include a lack of inter-sectorial coordination, a shortage of resources, low penalization of violators, a lack of adequate and continuous monitoring and implementation of smoke-free laws, and most importantly, low awareness among the general public regarding the health impact of tobacco and the laws that are related to it. According to the level of compliance that was found in our research, it is evident that there is a gap between policy action and public health gains. The variable rates of compliance in different types of public places may suggest that the factors that contributed to the success of one type of public place may be applicable to the success of another type of public place. However, in order to achieve a higher degree of compliance, it is required to employ certain tactics that are tailored to the nature of the public place in question. According to the findings of a study carried out in India, various facilitators and barriers to implementing smoke-free laws are present in particular types of public places [41]. It has been stated that public awareness of the law and strong support from the government in implementation, including the imposition of fines, are factors that facilitate the implementation of policies [41]. This has also been reported in a study in Nigeria [42]. Additionally, a series of sensitization programs for authorized officers and persons in charge of compliance, as well as public health campaigns to raise awareness of the public health benefits of the smoke-free law, are more likely to smoothen enforcement, reduce resistance among stakeholders, and eventually ensure compliance. A ‘tobacco awareness week’ or ‘tobacco awareness fortnight’ could be observed to raise public awareness. During these events, the general public would be educated about the policy and provisions, and a strict approach would be taken, in which the general public would be immediately fined, sending a message that the government is serious about the law [43]. Moreover, there should be a regular evaluation of the specific measures employed for smoke-free public areas at various locations, which may have a positive effect.

Studies conducted in India and Nepal reported that people were more likely to smoke if the owners/managers of the public places themselves smoked [22,32]. Various measures can be taken to discourage smoking by the owners/managers, which ultimately lead to a reduction in active smoking. According to the Smoking and Tobacco Products Usage (Control) (Amendment) Act, 2013, Bangladesh, the manager/owner of the institution is held accountable for the instances where the laws are violated. The managers and owners should be well trained and updated about the laws regarding smoking in public places, including the fines and punishment levied in the cases where these laws are breached.

Along with the robust implementation of the law, it is also necessary to make the public aware of the existence of such policies [41–43]. The accountable department can use mainstream media and social platforms to make sure the people know against smoking in public places. To reach the younger generations earlier, textbooks should be updated, and mandated classes should be well adapted to change the social norm.

The protection of public health requires a number of different interventions, one of which is a complete smoke-free policy. However, merely enforcing the legislation is not enough. If a smoke-free law is not accompanied by a robust enforcement system that has clearly defined regulations, then the law will not have the desired effect of lowering the number of people who use tobacco products and who are exposed to SHS [44,45]. As a result, the government of Bangladesh needs to move quickly in order to set up a coordinated enforcement system that will facilitate people to comply with the smoke-free laws. A combination of both strong legislative implementation to enhance compliance and improvement in social norms that deem smoking in public places harmful can contribute significantly to reducing second-hand smoke exposure.
