# Supplementary material for: Compliance with smoke-free legislation in public places: An observational study in a northeast city of Bangladesh
Source: PLoS One. 2023 Apr 26;18(4):e0283650. doi: 10.1371/journal.pone.0283650 (PMC10132694; doi:10.1371/journal.pone.0283650)
Supplement: Attachment — Submitted filename: Response to Reviewers_V-2.docx [file pone.0283650.s005.docx]

**Response to Reviewers**

We are giving thanks to the Academic Editor and the reviewer for their comments on the decision letter. However, we cannot agree with the comments from the Academic Editor and Reviewer-1. We have given a point-by-point response to the comments from the Academic Editor and Reviewer-1.

**Authors’ Responses to Academic Editor:**

**Review Comment #1:** Precisely, the response/rebuttal received against the original reviewers' comments are neither satisfactory nor in compliance with the journal requirements. In all, the revised manuscript fails to address the major concerns raised by the reviewers in their original evaluation of the manuscript.

**Reply from the authors:**

Thank you for your comment. The decision of rejection is very disappointing for us based on the comments from the Academic Editor and the Reviewer-1. We are not sure how our response/rebuttal against the original reviewers’ comments are neither satisfactory nor in compliance with the journal requirements. In the revised version-1, we addressed all the comments from reviewer-2. In the final decision letter, we did not see any comments from reviewer-2.

However, reviewer-1 did not reject our manuscript. In the first round of revision, reviewer-1 suggested us to follow one of his three suggested methods for average compliance calculation. We accepted the suggestion of reviewer-1 and updated our method of average compliance calculation. In the revised version of the manuscript, we kept two methods for average compliance calculation. The first method was according to the suggestion of reviewer-1. Based on the previous research and our own consensus, we followed the third suggested method of the reviewer and made some modifications to it as we thought that it would be more feasible for average compliance calculation. The second method is the most commonly used method for average compliance calculation though reviewer-1 opposed this method. The second method was used in the recently published similar articles in high-impact journals (Plos One, BMJ Tobacco Control etc.). We mentioned in the statistical analysis section that this method was used in previous similar research. So, we used this method. All our changes in the manuscript according to the reviewers' suggestions are clearly visible in the revised version (in track changes) of the manuscript. Moreover, in the Review Response Report, we gave necessary explanations on how we addressed all the comments of the reviewers.

**Authors’ Responses to Reviewer 1:**

**Review Comment #1:**  My comments are not addressed. Either should have corrected the overall compliance percentages or have given a justification of the method of average used.

**Reply from the authors:**

Thanks for your comments. The comment is disappointing for us. We have addressed the reviewer’s comments and followed the suggestions from the reviewer. Our changes and modifications can be found in the revised version-1 (please see the track changed copy of the revised version-1). Moreover, in the Review Response Report, we gave necessary explanations on how we addressed all the comments of the reviewers. For convenience to make adjudication, we are mentioning below again how we addressed the reviewer comments in revised version-1:

**The comment from the reviewer-1 was as follows:**

**The average percentages concept used in the analysis, cannot be considered correct. Few suggestions for overall percentages**

1. Summing up all compliance options with OR condition (for instance if for one case {absent of active smoking =1, presence of no smoking signage = 0, Display of no signage at entrance = 0, no smoking signage is in compliance with law = 1, Absence of cigarettes buts, bidi ends or ashes = 1, absence of smoking aids = 0} for overall compliance it will count "1" for that cases) The overall percentage will be calculated through that column of overall. this scenario will work if every option is given equal weight age.

2. If every option has different weightage then maximum of the weightage will be the final option.

3. A criterion can be made (e.g. if 4 of the 7 compliances are answered "yes" and 3 are answered "no", overall it will go to "yes" IF overall percentages are changed by using one of these methods then the manuscript is acceptable in my point of view. If some justification for using average percentages is provided by the author and is acceptable by the editorial board or by some other expert in the field that will be appreciable.

**Our reply was as follows:**

Thanks for raising this concern. However, this average percentage concept has been used in several previous research published in high-impact journals, including a recent publication. We have mentioned in the “Statistical Analysis” section of our manuscript that we have used this approach as it has been used in previous research. The references are the followings:

Goel S, Sharma D, Gupta R, Mahajan V. Compliance with smoke-free legislation and smoking behaviour: Observational field study from Punjab, India. Tobacco Control. 2018;27: 407–413. doi:10.1136/tobaccocontrol-2016-053559

Goel S, Ravindra K, Singh RJ, Sharma D. Effective smoke-free policies in achieving a high level of compliance with smoke-free law: Experiences from a district of North India. Tobacco Control. 2014;23: 291–294. doi:10.1136/tobaccocontrol-2012-050673

Basnet LB, Budhathoki SS, Adhikari B, Thapa J, Neupane B, Moses T, et al. Compliance with the smoke-free public places legislation in Nepal: A cross-sectional study from Biratnagar Metropolitan City. PLoS ONE. 2022;17. doi:10.1371/JOURNAL.PONE.0264895

However, we assume that the suggestions of the reviewer are also worthy, and it will improve the quality of the reporting of the manuscript. So, based on the reviewer’s suggestions and our consensus, we have also rated the compliance in the following ways:

For indoors, a location was given a rating of ‘good compliance’, ‘moderate compliance’ or ‘poor compliance" depending on the number of indicators it met: 5-7, 3-4, or 0-2 for good compliance, moderate compliance, or poor compliance, respectively. For outdoors, a location was leveled as ‘good compliance’, ‘moderate compliance', or ‘poor compliance’ if the place complied with 5-6 indicators, 3-4 indicators, or 0-2 indicators, respectively.

The interesting fact is that percentage of average/overall compliance, and moderate compliance (using the new approach) are almost identical in most cases.

**So, it is sorrowful to hear from the reviewer that we did not address the comments of the reviewer.**
